# Regadenoson blood flow in type 1 diabetes (RABIT_1_D Study)

**DOI:** 10.1186/1532-429X-16-S1-O59

**Published:** 2014-01-16

**Authors:** Daniel W Groves, Janet K Snell-Bergeon, Devavrat Likhite, Edward V DiBella, Marian J Rewers, Robert A Quaife

**Affiliations:** 1 Cardiology, University of Colorado, Denver, Colorado, USA; 2 Barbara Davis Center, Departments of Pediatrics, Preventive Medicine, and Biometrics, University of Colorado, Denver, Colorado, USA; 3 Radiology, Utah Center for Advanced Imaging Research, University of Utah, Salt Lake City, Utah, USA

## Background

Cardiovascular disease is the leading cause of mortality in type 1 diabetics (T1D). T1D patients have increased coronary artery calcification (CAC) compared to non-diabetics. We hypothesize that myocardial blood flow (MBF) reserve can be measured in long-standing T1D patients using regadenoson stress cardiac magnetic resonance (CMR) perfusion imaging and is a marker of extensive atherosclerotic disease associated with CAC.

## Methods

**Figure 1:**
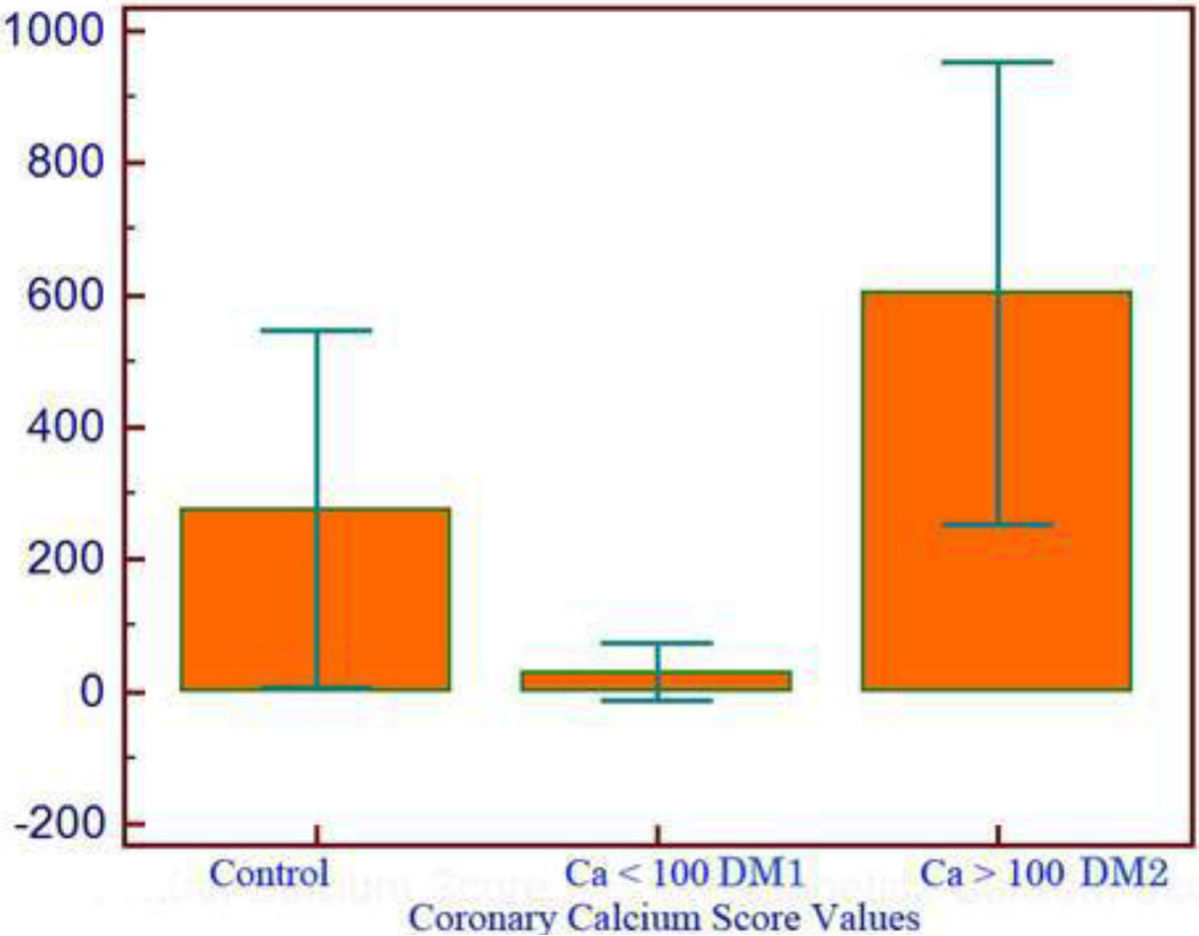
**Coronary artery calcium score by study group**.

RABIT_1_D is a sub-study of the Coronary Artery Calcification in Type 1 Diabetics (CACTI) study that established a cohort of 656 T1D patients and 764 non-diabetic controls that was followed for progression of CAC over 6 years with electron beam tomography. Non-diabetic controls with CAC > 100 (n = 4), T1D patients with CAC < 100 (n = 5, DM1), and T1D patients with CAC > 100 (n = 16, DM2) were recruited to complete sequential regadenoson stress nuclear imaging (MPI) and CMR perfusion imaging. CAC scores for the three groups are located in Figure 1. Patients with abnormal MPI were referred for invasive coronary flow reserve measurements in a cardiac catheterization lab. Twenty-three cardiac MRI studies were performed of which 20 were analyzable (n = 4 in control, n = 4 in DM1, n = 12 in DM2). Three studies were not analyzable due to poor image quality from motion, system error, and a patient dropping out of the study. CMR perfusion imaging was performed at rest followed by imaging during maximal vasodilation after 400 mcg injection of regadenoson. CMR images were acquired with a 4 channel phased array surface coil. Three simultaneous short-axis slices (basal, mid, and apical) were acquired during an initial bolus injection of gadolinium (ProHance, 0.05 mmol/kg at a rate of 5 mL/sec). SSFP imaging sequence was used with TR, TE, flip angle of 185 ms, 1.2 ms, 50 degrees, respectively, and inserted into a 330 × 380 matrix. Images were acquired using a field of view ranging from 280 to 400 mm. MBF reserve measurements were obtained from 6 regions within the mid-ventricular short axis slice using the Fermi deconvolution method.

## Results

**Figure 2:**
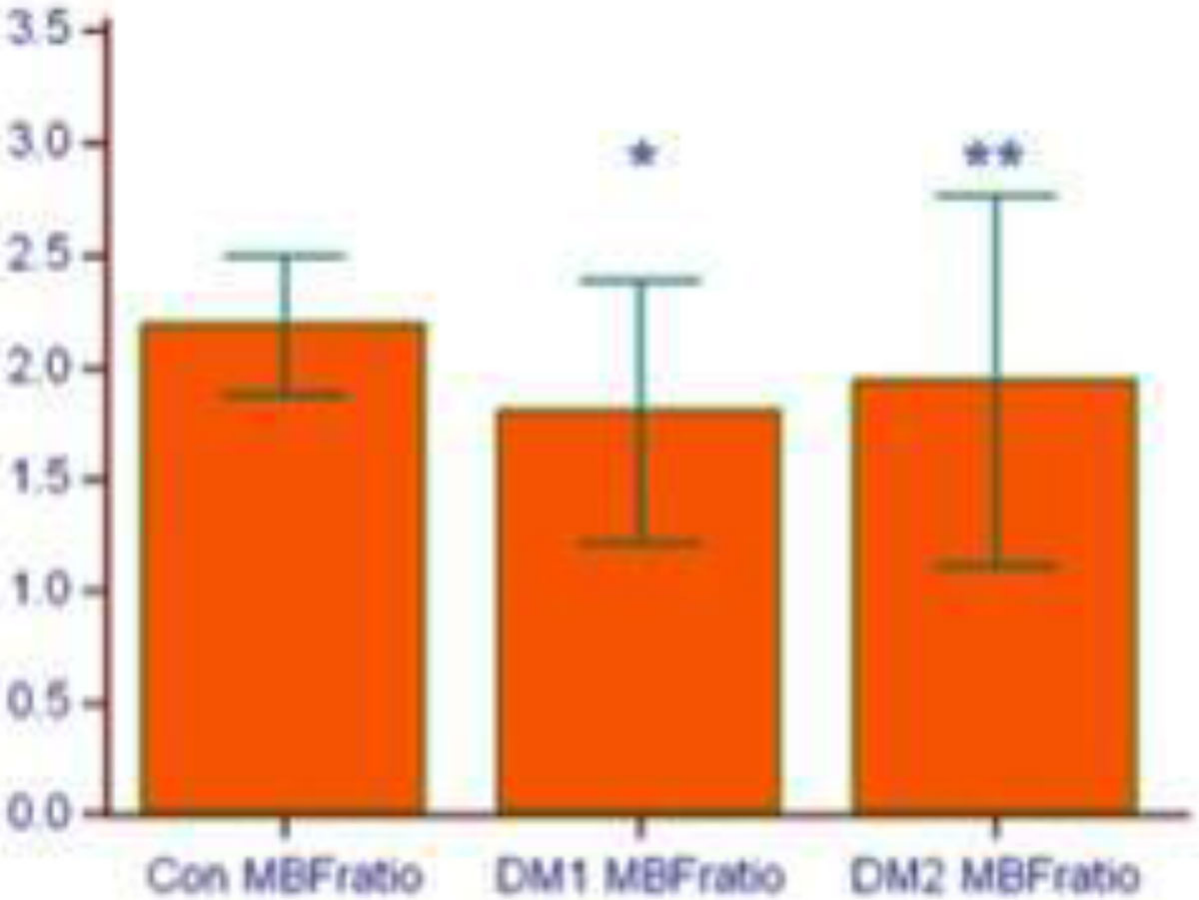
**Myocardial blood flow reserve by study group**.

MBF reserve mean value was 2.19 ± 0.3 for the moderate risk non-diabetic controls while MBF for the low and high risk T1D groups were 1.81 ± 0.6 and 1.95 ± 0.7, respectively. There was a significant difference between the controls and DM1 group (p = 0.001, *) and DM2 group (p = 0.007, **) demonstrated in Figure 2. No difference was noted between DM1 and DM2 groups (p = 0.067).

## Conclusions

MBF reserve was successfully measured in regadenoson CMR perfusion imaging studies. Regadenoson stress imaging was safe in the T1D patient groups. In this preliminary study, the impaired vasodilator reserve in T1D patients may indicate more extensive atherosclerotic disease than in moderate-risk controls with similar or higher CAC scores.

## Funding

The CACTI study was supported by National Institutes of Health National Heart, Lung, and Blood Institute grant HL61753. Funding for RABIT_1_D was provided by Astellas Pharma US Inc.

